# Association of Intimal Neovessels Noted by Optical Coherence Tomography with Cardiac Allograft Vasculopathy

**DOI:** 10.7759/cureus.7454

**Published:** 2020-03-29

**Authors:** Diljon S Chahal, Ravi Parikh, David Yoo, Temilolu Aje, Gautam Ramani, Mukta C Srivastava

**Affiliations:** 1 Interventional Cardiology, University of Maryland School of Medicine, Baltimore, USA; 2 Interventional Cardiology, Scripps Clinic, San Diego, USA; 3 Cardiology, Scripps Clinic, San Diego, USA; 4 Cardiology, LifeBridge Health System, Eldersburg, USA; 5 Cardiology, University of Maryland Medical Center, Baltimore, USA; 6 Interventional Cardiology, University of Maryland Medical Center, Baltimore, USA

**Keywords:** cardiac allograft vasculopathy, optic coherence tomography, intimal neovessels

## Abstract

Background

Cardiac allograft vasculopathy (CAV) is a leading cause of graft failure in cardiac transplant recipients. Progression of intimal thickening noted during routine surveillance intracoronary imaging is associated with the development of CAV. However, mechanisms of CAV development are poorly understood and targets for therapy modification remain elusive. We investigated the association of neovessels (INs) within the intima, noted by optical coherence tomography (OCT) during routine CAV surveillance imaging, with intimal thickening and co-incident CAV.

Methods

Coronary angiography and OCT images of 45 consecutive cardiac transplant recipients undergoing surveillance coronary imaging were reviewed. The presence of INs, defined as dark, tubular or rotund intimal structures, measuring 50-200 µm in diameter, noted in at least three OCT frames, was quantified. CAV diagnosis was determined by utilizing the International Society of Heart and Lung Transplant classification system. Demographic and clinical data was obtained by chart review. Significant associations between the presence of INs and CAV, intimal thickening, and demographic features were evaluated.

Results

INs were observed in 22/45 evaluated patients (49%), while angiographic CAV was observed in 24/45 patients, with a significant association noted between the presence of INs and CAV (*p* < 0.001). INs were also associated with increasing intimal thickness (*p* < 0.001), co-morbid hypertension (*p* = 0.010), and increasing transplant age (*p*= 0.002) on multivariate analysis.

Conclusion

INs are prevalent in cardiac transplant recipients and are significantly associated with CAV, increased intimal thickness, increasing transplant age, and co-morbid hypertension. Further investigation is warranted regarding the temporal relationship of IN development and the onset of CAV, as well as the mechanisms of IN development in this population.

## Introduction

The International Society for Heart and Lung Transplantation (ISHLT) reports that approximately 5,000 cardiac transplantations are performed annually at participating centers worldwide [1]. The long-term durability of cardiac transplant is limited by immunologic graft failure, termed cardiac allograft vasculopathy (CAV), which is a leading cause of death in cardiac transplant recipients who survive the first post-transplant year. CAV is a complex, immune-mediated inflammatory process that results in endothelial dysfunction and vascular injury. Diffuse fibro-proliferative concentric intimal hyperplasia ensues, leading to compromised coronary blood flow and ischemia, resulting in graft dysfunction, arrhythmias and sudden cardiac death [2].

Early diagnosis of CAV is challenging due to cardiac denervation leading to an absence of early clinical symptoms of ischemia. Routine coronary angiography with intra-vascular imaging to monitor intimal thickening has been utilized in CAV prognostication and is a mainstay of surveillance measures for CAV at many transplant institutions [3]. Mechanisms of CAV development are poorly elucidated and adequate targets for disease modification remain lacking.

Optical coherence tomography (OCT) is a light-based intra-vascular imaging modality with 10 to 15-μm resolution, accurately evaluating intimal thickening as well as delineating intimal structures such as neovessels (INs) [3]. Neovessels have been well described in native heart atherosclerosis and have been observed in accelerated CAV [1,4]. The role and incidence of INs in CAV, which may provide valuable insight into delineating a multi-factorial disease process, have not been elucidated. In this study, we evaluated the prevalence of INs in cardiac transplant patients and their association with angiographically diagnosed CAV.

## Materials and methods

Patient selection and interventional technique

This analysis included a retrospective review of 45 cardiac transplant recipients undergoing routine post-transplant coronary angiography and left anterior descending (LAD) vessel imaging with OCT between March 2013 and June 2015. After completion of diagnostic coronary angiography and typically after administration of intracoronary nitroglycerin (200-400 µg), a 2.7F OCT imaging catheter (Dragonfly-Duo, St. Jude Medical, St. Paul, MN) was advanced over a guide-wire into the mid-to-distal LAD. OCT images were obtained during continuous contrast injection (3-4.5mL/s, 12-15 mL total) at 300-500 pSI and 0 rate of rise. The total scan length was 50-75 mm acquired at a rate of 100 frames per second at a pullback speed of 25mm/sec. 

OCT analysis

OCT images were analyzed utilizing an offline review workstation (LightLab Imaging, St Jude Medical, USA) in a frame-by-frame fashion jointly by two operators, assessing for both the presence and severity of neovascularization within the intimal layer of the LAD. To distinguish neovessels from side-branches, an IN was defined as a dark, sharply delineated tubular or rotund structure within the intimal layer, measuring at least 50 µm, but no greater than 200 µm in diameter, and seen on at least three consecutive frames. For neovessels seen traversing the media into the intima, a progressive decrease in size was required to be classified as an IN. Maximal intimal thickening was measured utilizing the ruler too, from the lumen to the internal elastic membrane.

Angiographic analysis

Angiographic analysis was completed by two independent observers, utilizing the ISHLT nomenclature system to categorize disease as ISHLT CAV0 (no disease), ISHLT CAV1 (mild disease), ISHLT CAV2 (moderate disease), and ISHLT CAV3 (severe disease). Discrepancies between observers in CAV Grade classification were addressed by joint review, leading to consensus in all cases.

Statistical analysis

Demographic and co-morbid conditions were obtained by retrospective chart review. Patients were divided into two groups: those with and those without the presence of neovessels. Demographics, traditional clinical cardiovascular risk factors, transplant-related variables and medications, and echocardiographic imaging data were compared using a one-way ANOVA for continuous parametric variables and Kruskal-Wallis test for continuous non-parametric variables. Binary variables were compared across quintiles using the Pearson Chi-Square test. Multivariate analysis was performed using logistic regression analysis. Demographic and transplant-related factor-adjusted models were used to determine the risk of incident cardiac allograft vasculopathy. SPSS version 22 (IBM SPSS Statistics, IBM Corporation, Armonk, New York) was used for statistical analysis.

## Results

Participant characteristics and cross-sectional associations

OCT studies from 45 heart transplant recipients were included in the analysis. Among the population, 24 of 45 patients (53.3%) had incident CAV, most of which was grade I (Tables 1, 2).

**Table 1:** Baseline characteristics BMI, body mass index; OCT, optical coherence tomography; CMV, cytomegalovirus; CKD, chronic kidney disease as determined by chart review; LVEF, left ventricular ejection fraction; MIT, maximal intimal thickness; CAV, cardiac allograft vasculopathy; SD, standard deviation; IQR, interquartile range

Parameter	Neo+ (n=22)	Neo- (n=23)	P value
Age, mean (SD)	58.8 (12.1)	57.5 (14.8)	0.955
Male gender	19 (86.4%)	19 (82.6%)	0.728
Black race	6 (27.3%)	6 (26.1%)	0.928
BMI (kg/m^2^), median (IQR)	27.6 (24.0-2.1)	26.1 (23.8-29.4)	0.394
Transplant age, median (IQR)	6.3 (3.0-8.5)	2.0 (0.4-4.0)	0.002
Rejection	9 (42.9%)	9 (39.1%)	0.802
Ischemic cardiomyopathy (pre-transplant)	6 (27.3%)	7 (30.4%)	0.815
Medications at time of OCT			
Steroids	11 (50.0%)	13 (56.5%)	0.661
Cellcept	16 (72.7%)	18 (78.3%)	0.666
Tacrolimus	17 (77.3%)	19 (82.6%)	0.655
CMV history	4 (18.2%)	6 (26.1%)	0.524
Diabetes mellitus	10 (45.5%)	12 (52.2%)	0.652
Hypertension	22 (100.0%)	17 (73.9%)	0.010
Hyperlipidemia	19 (86.4%)	19 (82.6%)	0.728
Active smoking	1 (4.5%)	0 (0.0%)	0.301
CKD	6 (27.3%)	12 (52.2%)	0.088
LVEF post-transplant (%), median (IQR)	60 (55-65)	60 (55-65)	0.953
MIT(μm), median (IQR)	540 (420-765)	280 (210-370)	<0.001
CAV	18 (81.8%)	6 (26.1%)	<0.001
C4d+ > weak within 1 year of transplant	8 (40.0%)	11 (47.8%)	0.610

**Table 2:** CAV incidence and grade in the patient cohort CAV, cardiac allograft vasculopathy

CAV grade	Number of patients (n = 45)
0	21 (46.7%)
1	21 (46.7%)
2	0 (0.0%)
3	3 (6.7%)

Table 1 reflects the baseline demographic and clinical characteristics of the group divided by the presence or absence of INs. There were 22 patients with observed intimal neovessels on OCT (48.9%) with the majority having incident CAV (81.8%). The presence of neovessels was significantly associated with a history of hypertension, older transplant age, and higher maximal intimal thickness (MIT). However, neovessels were not associated with other traditional cardiovascular risk factors such as diabetes, smoking, prior stroke, peripheral vascular disease, chronic kidney disease, and immune-suppressive transplant medications.

 

Neovessels and incident CAV

**Figure 1:**
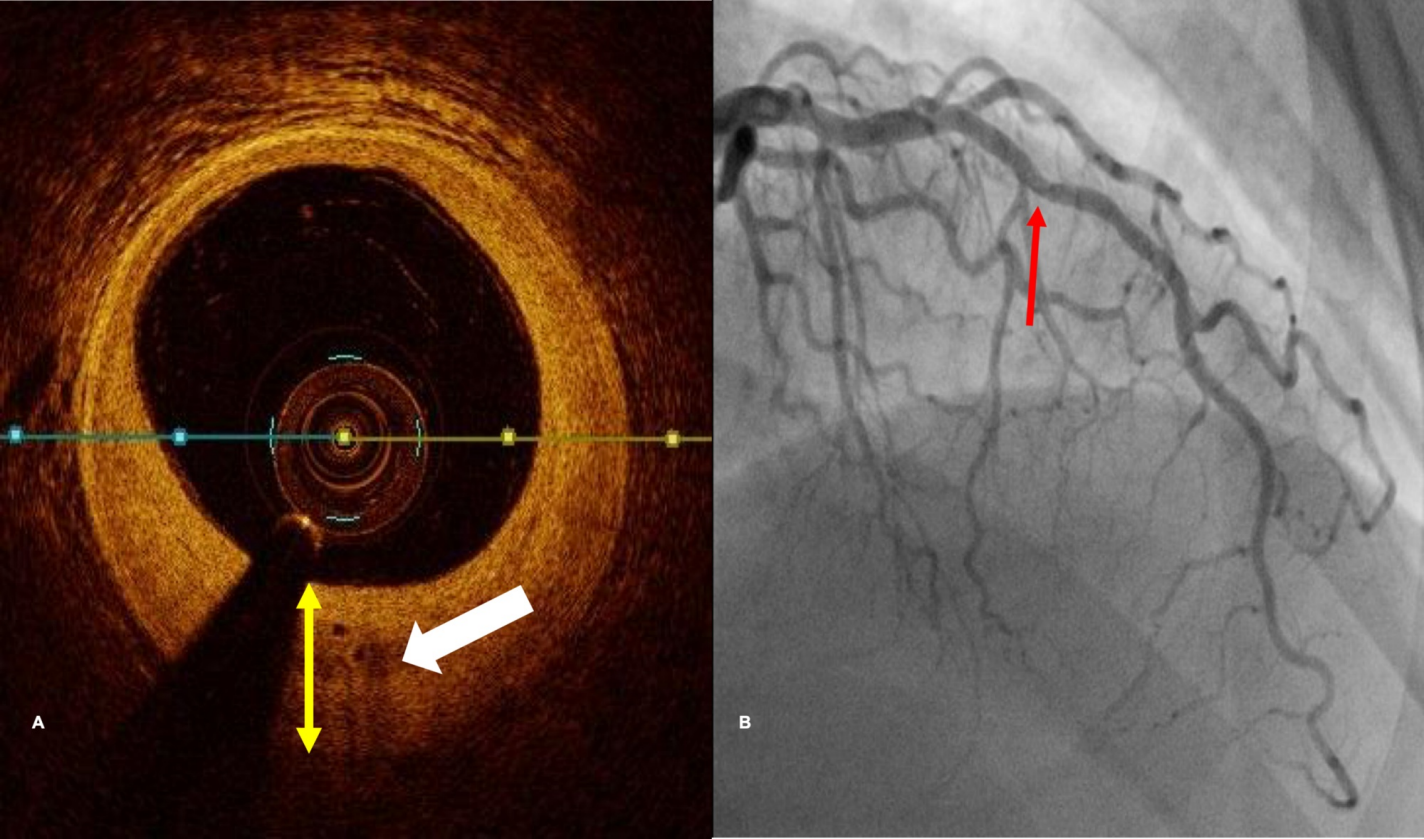
Correlation of angiographic CAV with intimal neovessels The area indicated by the red arrow on the angiogram (Panel B) is evaluated by optic coherence tomography on Panel A. The yellow double-headed arrow indicates intimal thickness. The heavy white arrows highlight intimal neovessels. CAV, cardiac allograft vasculopathy

Figure 1 shows a correlation of angiographic CAV with INs noted on OCT. There was over a 12-fold increased risk of incident CAV with the presence of neovessels (unadjusted odds ratio (OR) 12.8; 95% CI 3.1-53.2). These associations remained significant, though slightly attenuated, in the demographic-, transplant age-, and MIT-adjusted models (adjusted OR 7.8; 95% CI 1.3-49.1). Transplant age and MIT were independently associated with incident CAV (*p *= 0.03 for both variables).

## Discussion

Our study demonstrated: (1) INs, as visualized by OCT, are prevalent in heart transplant recipients; and (2) INs are significantly associated with CAV, hypertension, increasing transplant age, and maximal intimal thickness. In this analysis, we link intimal neovascularization observed by OCT to co-incident CAV, establishing an association between IN visualization and clinical outcomes. Importantly, we also provide further demonstration of IN associations noted in small series by other investigators [5-7].

Corroboration with prior series

In the largest study of prospective intravascular evaluation by OCT of cardiac transplant patients, Lerman *et al*. reported a prevalence of INs of 46% at greater than four years post-transplant, comparable to the prevalence in our series with a median transplant age of 6.3 years [5]. Ichibori *et al*. reported increased intimal neovascularization at greater than 1-year post-transplant, which corroborates our finding of a significant association of INs with increasing transplant age. We also similarly observed an association between increasing MIT and the presence of INs (*p* < 0.001) [6]. We did not observe a significant association between INs and recipient hyperlipidemia, diabetes or prior CMV infection, as described by other authors, but did note an association with recipient hypertension [6,8]. Given that traditional coronary artery disease risk factors and CMV infection are established risk factors for CAV, this discrepancy may be a result of our small sample size being underpowered to establish an association with multiple demographic features.

CAV and intimal neovascularization

CAV is a fibro-proliferative inflammatory process associated with significant cardiac events and graft failure. Landmark studies utilizing intravascular ultrasound (IVUS) for surveillance in cardiac transplant patients have demonstrated that an increase of intimal thickness greater than 0.5 mm from baseline evaluation to the first post-transplant year is associated with marked CAV progression and increased mortality [9-10]. However, advances in immune-suppression regimens since these studies have not led to improvements in the incidence and rate of progression of CAV, leading to the desire for more sensitive markers of CAV which could allow for earlier detection of the disease process [11]. OCT utilizes an optical analog of ultrasound and provides ten times greater spatial resolution compared to IVUS, allowing for more precise measurement of intimal thickening, as well as visualization of intimal structures such as neovessels and macrophages and delineation of plaque components, with the potential to provide further characterization and prognostication of CAV [5,12]. Our findings of a significant link between the observation of INs and the presence of CAV substantiate this potential for earlier detection of CAV which may inform modifications in immune-suppressive therapy to delay the progression of vasculopathy.

Intimal neovascularization is well described in native heart atherosclerosis, thought to be mediated by hypoxia-inducible factor 1-α (HIF-1α) activation in response to hypoxemia of the thickened intimal [13]. IN development in cardiac allograft vasculopathy may similarly result after initial intimal thickening resulting from immune-mediated endothelial insults. Ichibori *et al*. report a steep increase in intimal neovascularization in the early post-transplant phase, 8 weeks to 12 months after cardiac transplantation, suggesting that neovascularization may be mediated by an inflammatory post-transplant milieu [6]. Importantly, there is evidence to implicate neovessels in propagating vasculopathy, challenging their presence solely as markers of disease or as a compensatory response to endothelial disease. One proposed mechanism of neovessel-mediated plaque growth in ischemic atherosclerotic disease is through the creation of a pathway for leukocyte recruitment and lipid leak into intimal plaque which leads to a cascade of inflammatory immune responses with intimal proliferation [13]. Neovascularization has been directly linked to plaque progression and vulnerability in native atherosclerosis [14-15]. Further, in a rat model of cardiac transplant, administration of a regimen of TNP-470, an angiogenesis inhibitor, in conjunction with cyclosporine post-transplant, interrupted progression of CAV [16]. The identification of neovessels during surveillance imaging may thus provide both prognostic information as well as therapeutic targets in cardiac transplant patients [17].

 Our study is limited by a relatively small series of patients and the observational nature of the investigation. Baseline OCT evaluations were not available for all subjects, thus pre-existing donor INs and intimal thickening could not be accounted for. Long-term clinical follow-up data was not available; therefore, the prognostic implications of observing INs outside of a link to the development of CAV could not be commented upon. Finally, we could not account for the impact of included subjects having divergence of immune-suppressive regimens over time.

## Conclusions

In conclusion, INs are prevalent in cardiac transplant recipients and are significantly associated with CAV and intimal thickening as well as demographic features linked with unfavorable outcomes post-transplant. Further elucidation of the prognostic role of INs in CAV, as well as their role in the propagation of immune-mediated graft failure, may provide a therapeutic target for this disease process. Prospective serial evaluations would provide insight regarding timing and pathophysiology of the development of INs.
